# Emotional and cognitive processing of narratives and individual appraisal styles: recruitment of cognitive control networks vs. modulation of deactivations

**DOI:** 10.3389/fnhum.2012.00239

**Published:** 2012-08-25

**Authors:** Enrico Benelli, Erhard Mergenthaler, Steffen Walter, Irene Messina, Marco Sambin, Anna Buchheim, Eun J. Sim, Roberto Viviani

**Affiliations:** ^1^Department of Philosophy, Sociology, Education and Applied Psychology, University of PaduaPadua, Italy; ^2^Department of Psychosomatic Medicine and Psychotherapy, University of UlmUlm, Germany; ^3^Institute of Psychology, University of InnsbruckInnsbruck, Austria; ^4^Department of Psychiatry and Psychotherapy III, University of UlmUlm, Germany

**Keywords:** emotion, appraisal, appraisal styles, self-regulation, reading

## Abstract

Research in psychotherapy has shown that the frequency of use of specific classes of words (such as terms with emotional valence) in descriptions of scenes of affective relevance is a possible indicator of psychological affective functioning. Using functional magnetic resonance imaging (MRI), we investigated the neural correlates of these linguistic markers in narrative texts depicting core aspects of emotional experience in human interaction, and their modulation by individual differences in the propensity to use these markers. Emotional words activated both lateral and medial aspects of the prefrontal cortex, as in previous studies of instructed emotion regulation and in consistence with recruitment of effortful control processes. However, individual differences in the spontaneous use of emotional terms in characterizing the stimulus material were prevalently associated with modulation of the signal in the perigenual cortex, in the retrosplenial cortex and precuneus, and the anterior insula/ventrolateral prefrontal cortex. Modulation of signal by the presence of these textual markers or individual differences mostly involved areas deactivated by the main task, thus further differentiating neural correlates of these appraisal styles from those associated with effortful control. These findings are discussed in the context of reports in the literature of modulations of deactivations, which suggest their importance in orienting attention and generation of response in the presence of emotional information. These findings suggest that deactivations may play a functional role in emotional appraisal and may contribute to characterizing different appraisal styles.

## Introduction

Several systematic efforts have been made to empirically quantify change promoted by psychotherapy (Norcross, 2002; Lambert, 2004) and relate it to the mechanism through which treatment operates (Orlinsky et al., 2004; Kazdin, 2007). Empirical analyzes conducted on formal aspects of language have shown an association between transitions in phases of therapy and specific markers in the verbal production in the therapeutic session (Mergenthaler, 1996; Bucci, 1997). Two dimensions appear to be implicitly or explicitly involved in the identification of these markers, detecting changes in the emotional, cognitive, and behavioral regulation styles of patients. The first dimension gauges the presence or absence of emotional content in the verbal material by indexing words with emotional valence. The second dimension assesses the level of abstraction of lexical items. Abstraction is here a category specific to this area of research, characterizing the linguistic manifestations of cognitive-reflective processes by the incidence of abstract word form when describing emotional experience, often recognizable in German by their suffix (Mergenthaler, 1996, 2008; Buchheim and Mergenthaler, 2000).

An interesting aspect of these lexical markers is their capacity to elicit individual differences in samples of participants not undergoing psychotherapy, suggesting their more general importance in characterizing appraisal styles of possible relevance to psychological functioning. In particular, individuals differ in this respect when describing experiences of attachment, an issue of central clinical and psychotherapeutic importance (Ainsworth et al., 1978; Bowlby, 1982; Shaver and Clark, 1994; Mikulincer and Shaver, 2003; Bakermans-Kranenburg and van IJzendoorn, 2009). It has been shown that, when interviewed on the memory of past relationships and the access to attachment-related thoughts and feelings, healthy participants with insecure attachment patterns differ also in the propensity to use emotional and abstract linguistic markers to describe their experiences (Buchheim and Mergenthaler, 2000). In this context, scenes taken from a validated published instrument for the assessment of individual attachment patterns Adult Attachment Projective Picture System (AAP, George and West, 2001, 2011, 2012; Buchheim et al., 2006a,b) were chosen because of their effectiveness in eliciting discourse on affective themes, evoking themes such as loss, abandonment, separation, and death. In a recent neuroimaging study, these scenes, accompanied by a short textual description, were used to monitor progress in the long-term psychotherapy of depression (Buchheim et al., 2012). Because of the importance of emotional and cognitive access to attachment-relevant information to the emotional makeup of the individual, these results suggest that these linguistic markers may be sensitive indicators of individual styles in regulating access to affect generally. Hence, without claiming a definitive and exhaustive characterization of this issue, we use here the term *individual appraisal styles* operationally to refer to differences identified through the use of these markers.

Outside the field of psychotherapy research, different mental health outcomes have been associated with different habitual strategies of emotion regulation (Gross and John, 2003; Aldao et al., 2010). Neuroimaging studies have highlighted the importance of brain areas associated with cognitive control for emotion regulation processes, in particular the prefrontal lobes (Ochsner et al., 2004; Ochsner and Gross, 2005; Wager et al., 2008). More generally, theories of mental health and therapy that view failures of self-regulation as a lack of balance between automatic and controlled processes emphasize the importance of well-functioning prefrontal control for mental health (Phillips et al., 2003; DeRubeis et al., 2008). However, since attachment patterns shape interpersonal exchanges largely outside the control of the individual, one would expect access to affective information in this respect to be, at least in part, regulated in ways that are spontaneous, automatic, or procedural (Mauss et al., 2007). Interestingly, even in the absence of explicit instructions to regulate, individuals with different habitual emotion regulation strategies may present different brain activation patterns either at rest (Abler et al., 2008) or in the presence of emotional material (Drabant et al., 2009).

**Figure 1:**
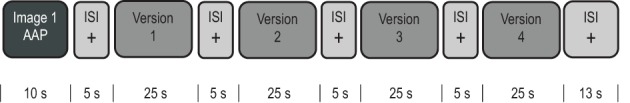
**Schematic depiction of a single trial.** Each of the eight trials (one for each of the eight scenes) contained four versions of the textual description of the same scene, representing each of the four conditions of the design, defined by the frequency of emotional or abstract words occurring in the text.

Our intention in the present study was to investigate the neural correlates of textual markers describing scenes of affective relevance. We used functional neuroimaging to identify the neural substrate of processing emotional or abstract descriptions of affectively evocative scenes, without giving any explicit instruction on the attitude to take relative to the emotional information present in the stimulus material. In the scanner, participants were exposed to four versions of the textual descriptions of eight situational or interactional attachment scenes (Figure 1), each crafted so as to systematically vary the presence of the linguistic markers identified by psychotherapy research (Mergenthaler, 2008). Participants were instructed to attentively read the textual description of the scenes.

To investigate individual differences in relating to the scenes depicted in the stimulus material, we asked participants to recount in writing the stories they had read after the scanning session had been completed. We reasoned that the spontaneous tendency of participants to use emotional or abstract terms in recounting the textual versions they had been exposed to in the scanner would contain information on their individual appraisal style. These recounted stories were scored in each participant by the frequency of the same linguistic markers that had been used to craft the story versions.

An issue we wanted to investigate was the extent to which the appraisal styles investigated here modulated prefrontal networks associated with emotion regulation, and cognitive control (Phillips et al., 2003; Ochsner and Gross, 2005; DeRubeis et al., 2008; Wager et al., 2008). A first question of interest therefore concerned the existence of increases in prefrontal activation in association with appraisal styles, even in the absence of an explicit instruction. Finding such an association would generalize the applicability of the prefrontal neurobiological markers of effective emotion regulation and mental health to the differences in the propensity to access emotional content captured by individual appraisal styles. We investigated this issue using a region of interest (ROI) approach, verifying the association of textual markers with the areas in the prefrontal cortex activated by the task. A second, more explorative analysis was undertaken to uncover correlates of appraisal styles in the whole brain. This second analysis allowed verifying the possible existence of brain networks associated with emotional appraisal and styles outside those associated *a priori* with cognitive control, and that might therefore uncover the neural substrates of the relatively spontaneous appraisal mechanisms examined here.

The present study differs in its scope and intention from previous studies investigating aspects of emotion appraisal of possible relevance for individual differences in accessing emotional information. First, we use material designed to evoke a reaction to complex but ecologically valid affective themes, despite using material that is primarily arousing as when using facial expressions or graphic pictures (Vuilleumier, 2005). Second, we were interested in investigating individual differences in the reaction to this material in the absence of an explicit instruction to appraise the material in a specific way or to focus on an alternative task. An important motivation for this approach is the possibility that an explicit instruction may influence aspects of individual appraisal that are best described as spontaneous, and that may be important in ordinary functioning.

## Materials and methods

### Participants

Healthy participants were recruited through local advertisements. All participants gave written informed consent. The study protocol was approved by the ethical committee of the University of Ulm, and was conducted in conformance with national legislation and the Code of Ethical Principles for Medical Research Involving Human Subjects of the World Medical Association. Three participants were excluded from analysis because of either excessive head movements or spikes identified in the fMRI data. The remaining 18 participants (6 male, 12 female, mean age 31, range 18–45) were right-handed German native speaker (Edinburgh Handedness Inventory, Oldfield, 1971). Current psychiatric illness was excluded by screening all subjects with scales evaluating anxiety (STAI, Spielberger et al., 1967; German version: Laux et al., 1981) and depression (CES-D, Radloff, 1977; German version: Hautzinger and Bailer, 1993) for scoring within the 95% percentile of the healthy population. Average STAI scores of participants were 35.9 (SD 7.3, range 25–43) for state and 37.1 (SD 9.8, range 27–52) for trait. CES-D scores averaged 12.1 (SD 8.53, range 4–25). Further exclusion criteria were use of drugs or psychoactive medication (by self-report).

### Stimulus material

The stimulus set consisted of eight attachment scenes from the AAP (George and West, 2001, 2012; Buchheim et al., 2006a,b; see also www.attachmentprojective.com) described by four versions of the same story with different levels of abstract and emotional words (adapted from Holzer, 2007, *Bindungsstil und emotional-kognitive regulation* [*Attachment Style and Emotional Cognitive Regulation*]. Unpublished Doctoral Dissertation, University of Ulm, retrieved from (http://vts.uni-ulm.de/doc.asp?id=5966); and Walter et al., 2006. *The relationship between linguistic emotion-abstraction patterns and attachment.* In Society for Psychotherapy Research, *Book of Abstracts: From Research to practice*, p. 180. Ulm: Ulmer Textbank) in a 2 × 2 factorial design. The four versions of the same scene were presented consecutively in a trial; hence, there were eight trials, one per scene (Figure 1). Each trial started with the brief presentation (10 s) of a picture depicting the scene elaborated on by the stories, and followed by the presentation of the four story versions in blocks lasting 25 s each (hence, the term block refers to the presentation of a story version, while the term trial refers to the presentation of the whole group of picture plus story version). Blocks were separated by pauses of 5 s in which participants viewed the fixation point. Trials were separated by pauses of 13 s, in which the same fixation point was displayed. Total scanning time was 19 min 33 s (more details on the preparation of the textual version and the presentation of stimuli are in the Appendix, including a representative example of the scenic prime and the textual descriptions). To avoid order effects, versions type sequence within trials was arranged in counterbalanced order according to a Latin squares scheme, which was permuted across subjects (Winer et al., 1991).

The text of the 32 versions was constructed following the therapeutic cycle model (TCM, Mergenthaler, 1996; see also Mergenthaler, 1985), a representative model based on empirical findings in the field of psychotherapy research, which identifies phases of psychotherapy through the frequency of emotional and abstract words in textual transcriptions from therapy sessions. TCM relies on computer-assisted content analysis based on dictionaries of words identified as markers for these dimensions of change cycles model software (CM). These dictionaries consist of words suitable to express affect, as verbs and adjectives, or forms expressing abstract concepts. The frequency of occurrence of the lexical markers that have been associated with four different phases of therapy are characterized by four patterns, which in the TCM terminology take the name of *relaxing* (when both emotion and abstraction levels are low), *reflecting* (high abstraction level), *experiencing* (high emotion level), and *connecting* (simultaneous presence of high emotion and abstraction levels). Valence of words in the versions where emotional words were present was matched for positive and negative terms (positive valence, mean 0.088; SD 0.027; min 0.034; max 0.13; negative valence, mean 0.086; SD 0.025; min 0.034; max 0.12; *t*_15_ = 0.203; *p* = 0.842). Table 1 summarizes the occurrence of these terms, as characterized by the TCM model, across the four story version types. Flesch Reading Ease levels of the stories ranged from 41 to 72. A repeated-measurement ANOVA (with scene as grouping factor) gave no significant effect of these levels for story type (*F*_(3, 21)_ = 1.56, *p* = 0.23).

**Table 1:** **Material**.

**Story version**	**Word count**		**Abstraction words (%)**		**Emotion words (%)**	
	**M**	**SD**	**M**	**SD**	**M**	**SD**
No emotional or abstract words (“relaxing”)	127.1	5.9	3.7	3.0	1.3	1.0
Abstract words (“reflecting”)	116.4	4.3	21.5	2.1	5.0	2.7
Emotional words (“experiencing”)	123.5	7.4	2.4	1.4	18.1	1.7
Emotional and abstract words (“connecting”)	117.5	6.4	14.2	3.7	16.7	3.2

Frequency of occurrence, in percent (M, mean and SD, standard deviation) of words classified as abstract or emotional in the TCM dictionary in the four story versions relaxing, reflecting, experiencing, and connecting.

### Procedure

Prior to scanning, participants were familiarized with the pictures of the eight scenes, each accompanied by a minimal version of the textual description (a short simple sentence naming what could be seen in the picture). The preliminary familiarization with the content of the scenes was meant to reduce the possible confound of novelty during scanning. In addition, the pictures and the minimal textual description used for familiarization were briefly presented (10 s) at the beginning of each trial during the scanning session to reduce the possible confound of memory load when reading the textual descriptions of the scenes. A few practice trials immediately after having been positioned in the scanner (executed while routine clinical images were taken to exclude pathology) allowed participants to familiarize with the task. The presentation of pictures of the scene prior to the texts of the story versions was indicated on two grounds. The first was the evocation of a situation that is potentially emotionally laden through material that has been validated in this respect (George and West, 2001; Buchheim et al., 2006a,b). The second was the recalling of the scene to which the textual descriptions referred before these descriptions were offered, avoiding the confounding of memory-retrieval effects at the presentation of the first story block. Participants were instructed to start reading the textual description over again if they finished before the end of the block, and were also informed that it was not important that they read the textual description to the end, but were asked to read all descriptions silently and carefully understand their content. We opted for presenting the material in written form, to avoid interference between auditory presentation and scanner noise.

After scanning, the pictures showing the scenes presented for familiarization and at the beginning of each trial were presented again, and participants were asked to write down what they remembered about the stories related to the scene. This allowed verifying that all participants had read the stories in the scanner, and provided individual regressors on the propensity to use abstract or emotional terms, as scored automatically with the CM software. For each participant, this software computes the relative frequency of emotional and abstract words, which were entered simultaneously in a multiple regression design matrix.

### Image acquisition methods, preprocessing, and statistical analysis

Magnetic resonance imaging (MRI) data were acquired with standard procedures detailed in the Appendix. Images were obtained using echo-planar imaging (EPI), in which one volume was acquired about every 1.5 s (TR = 1540 ms), resulting in the acquisition of 760 volumes over the study (about 16 volumes per block). The design matrix included the events of Figure 1, convolved with a canonical BOLD activation curve. Fixation was not modelled by regressors, thus implicitly constituting a baseline (through the inclusion of an intercept term in the design matrix). Three main contrasts of interest were defined *a priori*, constituted by the two factors (emotion and abstraction) and their interaction. Contrast effects were estimated at the first level and brought to the second level to account for subjects as a random factor. They were first tested in functional ROIs defined by the main activation, and then in a more comprehensive whole-brain analysis, in which the volume was not masked by regions of interests. Correction for multiple comparisons at voxel level was obtained through the false discovery rate (FDR) approach (Genovese et al., 2002), as implemented in the SPM5 package. ROIs were defined by the *p* ≤ 0.005 uncorrected threshold, but significant at the previous analysis at *p* ≤ 0.05 FDR. ROI analyses were conducted by averaging the signal in each ROI, and then using the averaged signal as the dependent variable in the model. Cluster-level tests (Friston et al., 1994) were conducted on clusters defined by the threshold of *p* = 0.005, uncorrected. Figures were generated using the freely available MRIcroN software (http://www.sph.sc.edu/comd/rorden/mricron/), which also provided the T1-weighted image for overlays and the anatomical and Brodmann area (BA) labels (Tzourio-Mazoyer et al., 2002).

Statistical analysis of data other than neuroimaging was carried out with the freely available package R (The R Foundation for Statistical Computing, www.r-project.org, Vienna, Austria).

## Results

### Behavioural data

To investigate the individual differences in the tendency to adopt emotional or abstract renditions of the AAP images, we analyzed the written narratives produced in the post scanner task using standard CM software. The rate of use of emotional words in participants was 7.1% (SD 1.1), ranging from 5% to 9%. The rate of use of abstract words was 6.8% (SD 1.9), and ranged from 3% to 10%.

### Neuroimaging data

To investigate the effect of linguistic markers in the stimulus set on areas associated with cognitive control, we first identified the areas activated by the reading task (Just et al., 1996), and looked at their modulation by these markers using a functional ROI approach. Three main contrasts of interest were defined *a priori*, constituted by the two factors (emotion and abstraction) and their interaction. This analysis was followed by a more comprehensive whole-brain search of the effect of emotion and abstraction on signal levels, where the data were examined on a more explorative basis. Finally, we looked at the influence of individual differences in the propensity to recount in writing these scenes with emotional and abstract words. Unless otherwise specified, all significance levels are corrected for the whole brain volume at FDR.

### Effect of main task and functional ROI analysis

**Figure 2:**
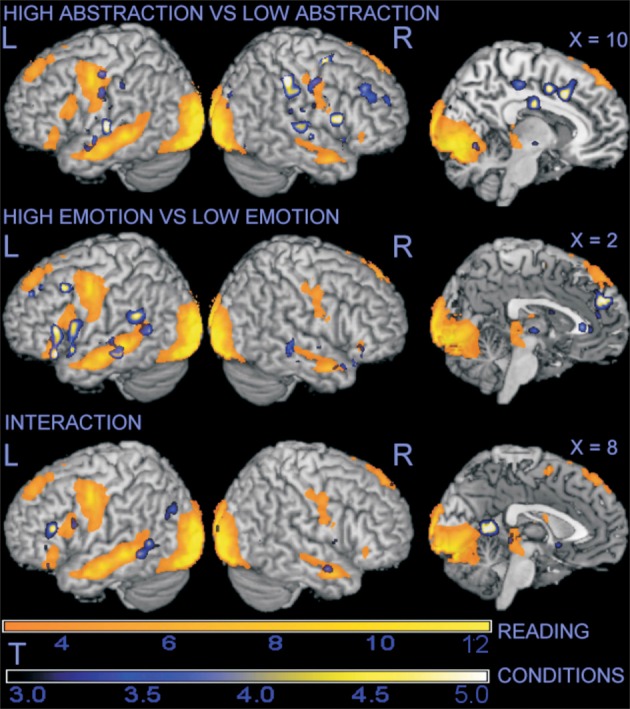
**Surface rendering projection of statistical maps associated with contrasts in the whole-brain analysis.** The large yellow/orange areas illustrate the activation elicited by the main task of reading stories, irrespective of content. Modulation of signal by abstraction, emotion, and their interaction is superimposed in blue/yellow. For illustration purposes, statistical maps were thresholded at *p* = 0.005, uncorrected.

Areas associated with reading, irrespective of story type, were identified by the contrast reading vs. fixation (see the areas in yellow/orange in any row of Figure 2). This contrast elicited a well-known activation pattern that included extensive occipito-parietal areas (BA17/18) and a predominantly left-lateralized network including the middle temporal gyrus (BA21), and parts of the prefrontal cortex extending from the dorsolateral (DLPFC, BA6) through the mediolateral (MLPFC, BA44/48) to the ventrolateral region (VLPFC, BA38/45; Vigneau et al., 2006; Awad et al., 2007; Yarkoni et al., 2008). These findings were accompanied by analogous, but less extensive, activations on the right (see Table 2 for details). On the medial line, there was some significant activation in the supplementary motor area on the left (BA6), and much more anteriorly in the superior frontal gyrus (SFG, BA9).

**Table 2:** **Main task**.

**Brain region**	**MNI Coord. (mm)**	***t***	***p* (uncorr., two-tailed)**	***p* (FDR corr., two tailed)**	**Cluster size (voxels)**	***p* (cluster corr., two-tailed)**
Reading vs. fixation						
L Occipital med/sup, Lingual (BA18/19)	−16, −94, −14	27.16	<0.001	<0.001	11014	<0.001
L middle temporal (BA21)	−58, −36, −2	12.43	<0.001	<0.001	2099	<0.001
L Hippocampus (BA20/27)	−20, −26, −8	11.50	<0.001	<0.001	1341	<0.001
L Precentral (BA6, DLPFC L)	−52, −4, 52	10.86	<0.001	<0.001	1531	<0.001
R Middle temporal (BA21)	56, 2, −22	7.60	<0.001	<0.001	628	0.006
L Supplementary motor area (BA6)	−6, 2, 64	6.55	<0.001	<0.001	77	>0.5
L Frontal superior (BA9, SFG)	−12, 48, 44	6.27	<0.001	<0.001	300	0.22
L Frontal inferior (BA44/48, MLPFC L)	−44, 14, 22	5.84	<0.001	<0.001	595	0.008
L Frontal inferior (BA38/45, VLPFC L)	−54, 30, −4	5.63	<0.001	<0.001	161	>0.5
L Putamen/Pallidum	−20, −6, 10	4.81	<0.001	0.004	229	0.54
R Precentral (BA4, DLPFC R)	58, −10, 50	4.74	<0.001	0.004	252	0.40
R Frontal inferior (BA45, VLPFC R)	58, 30, 0	4.09	0.001	0.012	24	1.00
Fixation vs. reading						
L Occipital middle (BA39)	−42, −86, 24	16.33	<0.001	<0.001	3557	<0.001
L Parietal inferior (BA40)	−52, −42, 40	13.09	<0.001	<0.001	s.c.	
L Fusiform (BA37)	−30, −54, −4	10.53	<0.001	<0.001	s.c.	
L Calcarine (BA18)	−14, −64, 18	9.23	<0.001	<0.001	s.c.	
L Cuneus (BA18)	−12, −70, 30	8.94	<0.001	<0.001	s.c.	
L Lingual (BA37)	−28, −48, −6	8.69	<0.001	<0.001	s.c.	
L Angular (BA39)	−58, −58, 36	8.21	<0.001	<0.001	s.c.	
L Temporal middle (BA22)	−58, −50, −16	7.94	<0.001	<0.001	s.c.	
L Temporal inferior (BA20)	−56, −46, −16	7.86	<0.001	<0.001	s.c.	
L Precuneus (BA7)	−6, −68, 36	6.76	<0.001	<0.001	s.c.	
L Temporal superior (BA48)	−56, −28, 22	5.57	<0.001	<0.001	s.c.	
L Hippocampus (BA20)	−38, −18, −10	13.72	<0.001	<0.001	24915	<0.001
R Temporal inferior (BA20)	56, −40, −18	13.61	<0.001	<0.001	s.c.	
L Caudate (BA25)	−8, 14, −8	13.34	<0.001	<0.001	s.c.	
L Frontal middle (BA44)	−38, 26, 36	13.21	<0.001	<0.001	s.c.	
L Cingulum anterior (BA24)	−6, 36, 18	12.96	<0.001	<0.001	s.c.	
R Hippocampus (BA20)	38, −12, −12	12.57	<0.001	<0.001	s.c.	
L Putamen (BA11)	−18, 18, −2	12.36	<0.001	<0.001	s.c.	
R Parietal inferior (BA40)	42, −52, 42	11.98	<0.001	<0.001	s.c.	
L Caudate	−14, 14, 2	11.52	<0.001	<0.001	s.c.	
R Caudate	14, 16, 4	11.43	<0.001	<0.001	s.c.	
R Temporal middle (BA39)	42, −70, 16	11.34	<0.001	<0.001	s.c.	
R Cingulum anterior (BA24)	6, 32, 20	11.14	<0.001	<0.001	s.c.	
L Insula (BA48)	−26, 18, −10	11.00	<0.001	<0.001	s.c.	
L Cingulum middle (BA23)	−6, −26, 36	10.79	<0.001	<0.001	s.c.	
R Fusiform (BA37)	32, −50, −6	10.77	<0.001	<0.001	s.c.	
R Frontal inferior orbitalis (BA38)	30, 24, −22	10.51	<0.001	<0.001	s.c.	
R Supramarginal (BA2)	58, −30, 36	10.40	<0.001	<0.001	s.c.	
L Pallidum	−8, 2, −2	10.33	<0.001	<0.001	s.c.	
R Frontal middle (BA45)	44, 44, 4	10.31	<0.001	<0.001	s.c.	
L Cingulum anterior (BA24)	−6, 20, 26	10.20	<0.001	<0.001	s.c.	
R Temporal superior (BA38)	58, 0, −4	4.23	0.001	0.004	10	1.0

Abbreviations: L left R right BA Brodmann area MNI Montreal Neurological Institute p (uncorr., two-tailed) significance levels, uncorrected p (FDR corr., two-tailed) significance levels at voxel level, false discovery rate p (cluster corr., two-tailed) significance levels cluster-level correction.

Cluster size in voxels of 2 × 2 × 2 mm. Reported are only cluster peaks significant at the level p < 0.05, false discovery rate. s.c., same cluster.

Based on this preliminary analysis, we defined functional ROIs in the prefrontal cortex to detect regions that, being possible neural substrates of processes responsible for the coordination of mental resources and selection of information, may characterize control processes specifically activated by the characteristics of the text. There were four such clusters: in the dorsolateral (DLPFC, BA6), mediolateral (MLPFC, BA44-48), and ventrolateral prefrontal cortex (VLPFC, BA38/45), and in the superior frontal gyrus (SFG, BA9).

The first contrast identified in the ROIs the effect of abstraction in the narratives, revealing a positive modulation of the DLPFC regions bilaterally (Table 3, top row). The second ROI contrast addressed the effect of emotion, and revealed significant signal increase in the VLPFC on the left and in the SFG (Table 3, middle row). The third and final ROI contrast tested the modulation of prefrontal activation that was specific to the simultaneous presence or absence of both emotional and abstract linguistic markers (Table 3, bottom row). This contrast reached trend significance in the left MLPFC.

**Table 3:** **Functional ROI analysis**.

	**DLPFC L**	**MLPFC L**	**VLPFC L**	**DLPFC R**	**VLPFC R**	**SFG**
High vs. low abstraction	*t* = 2.33^*^, *p* = 0.02	*t* = −0.59, *p* = 0.70	*t* = 0.22, *p* = 0.41	*t* = 2.73^*^, *p* = 0.01	*t* = −0.31, *p* = 0.6	*t* = −0.14, *p* = 0.56
High vs. low emotion	*t* = −0.99, *p* = 0.83	*t* = −1.18, *p* = 0.87	*t* = 2.60^*^, *p* = 0.01	*t* = 0.12, *p* = 0.45	*t* = 2.28^*^, *p* = 0.02	*t* = 1.98^*^, *p* = 0.03
Interaction	*t* = −0.06, *p* = 0.52	*t* = 1.94^*^, *p* = 0.03	*t* = 1.38, *p* = 0.09	*t* = 0.60, *p* = 0.28	*t* = −0.48, *p* = 0.68	*t* = −1.39, *p* = 0.91
Cluster size	1531	595	161	252	24	300

Abbreviations: L left R right DLPFC Dorsolateral Prefrontal Cortex MLPFC Mediolateral Prefrontal Cortex VLPFC Ventrolateral Prefrontal Cortex SFG Superior Frontal Gyrus.

Significance values are one-sided. The asterisk“

* ” marks results significant at p = 0.05, one sided. Cluster size in 2 × 2 × 2 mm voxels.

The regression of the contrast signal in these ROIs on individual differences in the use of emotional or abstract words in the post-scan written recounting of the scenes was not significant.

### Whole-brain analysis

#### Abstraction

In the second part of the analysis, we extended the exploration of the effects of the contrasts of interest to the whole brain. Beside the modulation of activation in DLPFC bilaterally detected in the ROI analysis, the contrast on the presence of abstraction in the text material revealed the additional involvement of the dorsal anterior cingulus (dorsal ACC, BA32), the bilateral superior temporal gyrus (BA48), and, in the right hemisphere, the temporo-parietal junction (BA2/40) and the inferior frontal gyrus (BA45/47; see Figure 2, top row, and Table 4 for details). Further analysis revealed that these areas had different activation status in the main task. The dorsal ACC was significantly activated (*t* = 6.55, *p* < 0.001), as was the inferior frontal gyrus, albeit more weakly (*t* = 2.6, n.s.). The superior temporal gyri and the temporo-parietal junction were only weakly, and non-significantly, affected by the main task (*t*-values around −1.5/−1.9). Regression of the signal estimated by this contrast on individual differences in the use of abstract words in the post-scan written recounting of the scenes gave no significant results. In summary, whole brain analysis showed activation of the anterior medial prefrontal cortex, and involvement of the right hemisphere, particularly of the right temporo-parietal junction.

**Table 4:** **High vs. low abstraction, whole brain analysis**.

**Brain region**	**MNI Coord. (mm)**	***t***	***p* (uncorr.)**	***p* (FDR corr.)**	**Cluster size (voxels)**	***p* (cluster corr.)**
R Middle cingulum (BA32)	14, 16, 40	7.50^*^	<0.001	0.028	717	<0.001
L Superior temporal (BA48)	−54, −18, 6	7.28^*^	<0.001	0.028	122	>0.5
R Superior temporal (BA48)	50, −18, 6	7.13^*^	<0.001	0.028	1148	<0.001
L Cerebellum (BA18)	−12, −68, −20	6.56^*^	<0.001	0.028	190	0.23
R Inferior parietal (BA2/40)	48, −36, 48	6.54^*^	<0.001	0.028	309	0.04
L Superior temporal (BA41/21)	−44, −36, 12	6.18^*^	<0.001	0.028	302	0.04
R Precentral (BA6)	58, 10, 16	5.55^*^	<0.001	0.034	149	0.43
R Inferior frontal Triangularis (BA45/47)	40, 34, 0	5.42^*^	<0.001	0.036	593	0.001
L Lingual (BA19)	−20, −54, −8	5.33^*^	<0.001	0.040	58	>0.5
L Insula (BA48/20)	−40, 0, −8	5.12^*^	<0.001	0.043	278	0.06
R Middle cingulum (BA23)	8, −12, 30	5.02^*^	<0.001	0.044	82	>0.5
R Cuneus (BA19)	14, −74, 40	4.57	<0.001	0.056	129	>0.5
R Middle cingulum (BA23)	10, −26, 48	4.52	<0.001	0.059	172	0.30
R Insula (BA48)	36, −22, 8	3.84	0.001	0.091	56	>0.5
L Postcentral (BA3)	−36, −20, 40	3.82	0.001	0.092	56	>0.5
R Postcentral (BA4)	56, −12, 50	3.79	0.001	0.094	61	>0.5
R Postcentral (BA3)	46, −22, 40	3.78	0.001	0.095	74	>0.5
L Postcentral (BA3)	−54, −14, 38	3.58	0.001	0.107	76	>0.5

Abbreviations: L left R right BA Brodmann area MNI Montreal Neurological Institute p (uncorr.) significance levels, uncorrected p (FDR corr) significance levels at voxel level, false discovery rate p (cluster corr.) significance levels cluster-level correction.

Cluster size in voxels of 2 × 2 × 2 mm. The asterisk“

* ” marks results significant at FDR voxel level or cluster level, p ≤ 0.05. Reported are clusters of extent of 50 voxels or larger.

#### Emotion

In the contrast opposing text with high vs. low emotional content, the whole-brain analysis revealed positive associations with emotional content in the perigenual portion of the ACC (BA11/25), an area that was deactivated in the main contrast of the reading task (*t* = −9.09, *p* < 0.001). In the temporal lobes, the presence of emotional material was positively associated with the signal in areas activated by the main task. While more marked on the left, this modulation was present also on the right (Figure 2, middle row). The presence of emotional content was also associated with an activation of the amygdale that extended into the ventral striatum bilaterally (see Table 5 for details).

**Table 5:** **High vs. low emotion, whole brain analysis**.

**Brain region**	**MNI Coord. (mm)**	***t***	***p* (uncorr.)**	***p* (FDR corr.)**	**Cluster size (voxels)**	***p* (cluster corr.)**
L Superior medial frontal (BA25/24/32/9/8)	2, 46, 30	8.57^*^	<0.001	0.018	177	0.30
L Inferior frontal (BA47/45/48)	−44, 26, −14	6.96^*^	<0.001	0.050	256	0.09
L Middle frontal (BA9)	−44, 18, 48	5.47	<0.001	0.135	53	>0.5
L Middle temporal (BA20/21/22/48)	−52, −26, 2	5.43^*^	<0.001	0.135	359	0.02
R Middle temporal (BA21)	56, −34, −8	5.26	<0.001	0.149	286	0.06
L Supramarginal (BA42)	−62, −46, 26	5.22	<0.001	0.153	199	0.22
R Caudate	14, 10, 16	4.86	<0.001	0.204	84	>0.5
L Superior temporal pole (BA48)	−48, 10, −8	4.84^*^	<0.001	0.204	302	0.05
L Anterior cingulum (BA11/25)	−4, 34, 0	4.79	<0.001	0.204	102	>0.5
L Thalamus	−4, −14, 2	4.63	<0.001	0.204	60	>0.5
R Amygdala/ventral striatum	24, 4, −10	4.33	<0.001	0.204	107	>0.5
L Amygdala/ventral striatum	−22, 6, −10	4.20	<0.001	0.207	71	>0.5

Abbreviations: L left R right BA Brodmann area MNI Montreal Neurological Institute p (uncorr.) significance levels, uncorrected p (FDR corr) significance levels at voxel level, false discovery rate p (cluster corr.) significance levels cluster-level correction.

Cluster size in voxels of 2 × 2 × 2 mm. The asterisk“

* ” marks results significant at FDR voxel level or cluster level, p ≤ 0.05. Reported are clusters of extent of 50 voxels or larger.

**Figure 3:**
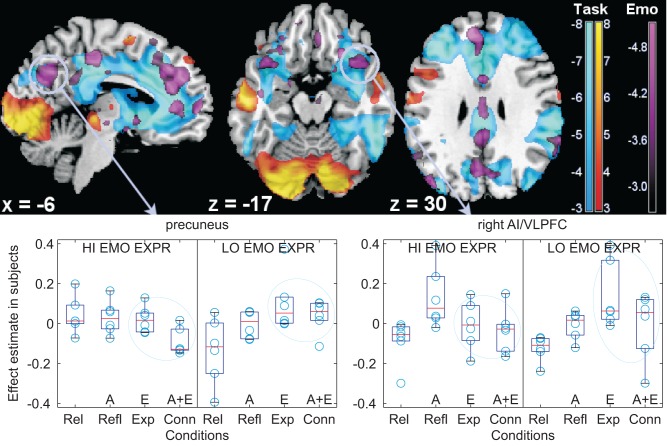
**Individual differences in the use of emotional words. Top:** modulation of deactivations by individual differences in the tendency to use emotional words in the medial aspect of the cortex (left), in the orbitofrontal cortex (centre), and at the level of the angular gyrus (right), shown as parametric maps of *t*-values overlaid on a template T1-weighted brain. Activations and deactivations of the main reading task are displayed in yellow/orange and light blue, respectively, thresholded for display purposes at *p* = 0.005, uncorrected. In violet, the modulation by individual differences, at threshold *p* = 0.01, uncorrected, of the emotional contrast. The left side of the transversal slices is on the left. **Bottom:** box-plots of signal differences between conditions, displayed separately in subjects with high and low use of emotional words (“HI EMO EXPR”, “LO EMO EXPR”) at the follow-up post-scan recounting of the stories (for illustration purposes, the first and last thirds of a tertile split on the emotional words use scores were used). Here and in the following boxplots, data were centered. Data are divided according to textual description type, in the TCM terminology: Rel (relaxing) contains few emotional or abstract words, Refl (reflecting) is rich in abstract words (“A” on the *x*-axis), Exp (experiencing) is rich in emotional words (“E” on the *x*-axis), and Conn (connecting) contains both emotional and abstract words (“A + E” on the *x*-axis). The light blue ovals highlight the textual version with emotional words, which differ in the groups with high and low use of emotional words at recounting the scenes. AI/VLPFC: anterior insula/ventrolateral prefrontal cortex.

**Figure 4:**
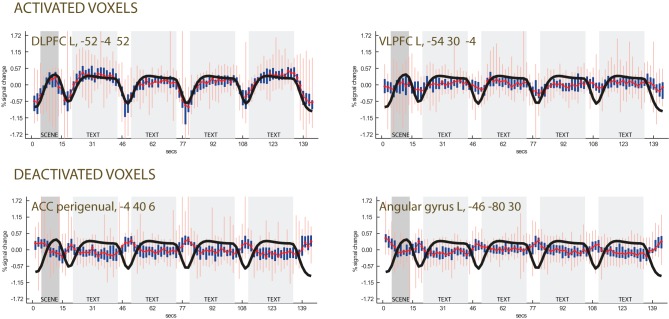
**Time-course plots in selected voxels (centered data). Top row:** activated voxels in the left DLPFC and VLPFC. **Bottom row:** deactivated voxels selected from the regression of the individual differences in the use of emotional terms, showing relative activation during fixation. The black line is the regressor obtained by convolving the task with a canonical BOLD curve, which tracks activation due to presentation of the scene (in dark gray background) and the textual descriptions (light gray background). The white background corresponds to the fixation point. The red line is the median signal. Thick blue lines show variation intervals at the 25th and 75th percentile of the data. Light red lines show the same variation at the 2.5th and 97.5th percentiles.

Regression of the signal estimated by this contrast on individual differences in the use of emotional words in the post-scan written recounting of the scenes revealed an association between the tendency to use emotional words when recounting the scenes and activation in the perigenual area (BA10/11/24/32), in the posterior cingulus/precuneus and retrosplenial cortex (BA30/4), the left angular gyrus (BA39) and in the anterior insula/VLPFC (BA38/47/45; see Figure 3 and Table 6). *Post-hoc* analysis revealed that, with the exception of the retrosplenial cortex, these areas were deactivated in the main task (*t*-values ranging from −6 to −9, all significant *p* < 0.02; see comparative plots of activations and deactivations of the perigenual ACC and the left angular gyrus in Figure 4). The considerable spatial overlap between task deactivation and the correlation with individual differences is visible in Figure 3 (top row). In the box plots (same Figure 3, bottom row), one can see that signal levels were modulated across story types in participants who later used few emotional words in recounting the stories, with text containing emotional words being associated with a less marked deactivation in these participants. In contrast, in participants that used comparatively more emotional words the deactivation across story types was much more similar.

**Table 6:** **Regression of emotional contrast on individual scores in use of emotional words in recounting the stories in writing**.

**Brain region**	**MNI Coord. (mm)**	***t***	***p* (uncorr.)**	***p* (FDR corr.)**	**Cluster size (voxels)**	***p* (cluster corr.)**
L Precuneus (BA30)	−2, −48, 18	−8.24^*^	<0.001	0.059	1330	<0.001
L Middle cingulum (BA4)	−4, −28, 50	−7.56^*^	<0.001	0.061	s.c.	
L Frontal superior medial (BA10)	−6, 60, 4	−7.14	<0.001	0.061	108	0.48
R Putamen/Pallidum	18, 6, −8	−7.06	<0.001	0.061	133	0.34
L Front. Sup. Med./Ant. Cingulum (BA32)	−10, 44, 26	−6.78^*^	<0.001	0.061	601	<0.001
L Angular (BA39)	−46, −80, 30	−6.27^*^	<0.001	0.069	335	0.03
R Insula (BA38)	38, 14, −16	−5.94	<0.001	0.071	152	0.26
L Anterior cingulum (BA11/25/32)	−4, 40, 6	−5.67	<0.001	0.082	258	0.06
R Middle frontal (BA9)	38, 26, 50	−5.51	<0.001	0.084	73	>0.5
R Inferior/Superior frontal orbitalis (BA11)	22, 28, −14	−5.34	<0.001	0.092	82	>0.5
L Inferior/middle frontal orbitalis (BA47)	−44, 48, −12	−5.29^*^	<0.001	0.092	467	0.003
L Caudate/pallidum/putamen	−8, 8, −6	−5.29	<0.001	0.092	133	0.34
R Inferior frontal triangularis (BA44)	58, 20, 22	−3.82	0.001	0.093	56	>0.5

Abbreviations: L left R right BA Brodmann area MNI Montreal Neurological Institute p (uncorr.) significance levels, uncorrected p (FDR corr) significance levels at voxel level, false discovery rate p (cluster corr.) significance levels cluster-level correction.

Cluster size in voxels of 2 × 2 × 2 mm. The asterisk“

* ” marks results significant at FDR voxel level or cluster level, p ≤ 0.05. Reported are clusters of extent of 50 voxels or larger. s.c.: same cluster.

In the opposite direction, increased use of emotional words in recounting stories in writing was not associated with significant modulation of the contrast effect. In the limbic system, a weak association was present in the anterior hippocampus on both sides, extending into the amygdala (*x*, *y*, *z*: −32, −6, −24, BA20; *t* = 3.39, *p* = 0.002, uncorrected;* x*, *y*, *z*: 30, −6, −22, BA20; *t* = 3.37, *p* = 0.003, uncorrected).

In summary, whole-brain analysis of the effect of emotion detected modulation of lateral temporal and left prefrontal activation and limbic system activation in the bilateral amygdalar complex. Emotion also modulated deactivation in the medial aspect of the perigenual cortex, correlating with individual tendencies to use emotional words in recounting the stories. Individuals who used less emotional words to later recount the scenes deactivated less in the presence of emotional words, accounting for relative activation of these deactivated areas in the emotional contrast. An association between individual tendencies to use less emotional words and effect of emotional words was also detected in the posterior cingulated/precuneus and in a localized region in the inferior frontal gyrus, extending into the adjacent orbitofrontal cortex.

#### Interaction

**Figure 5:**
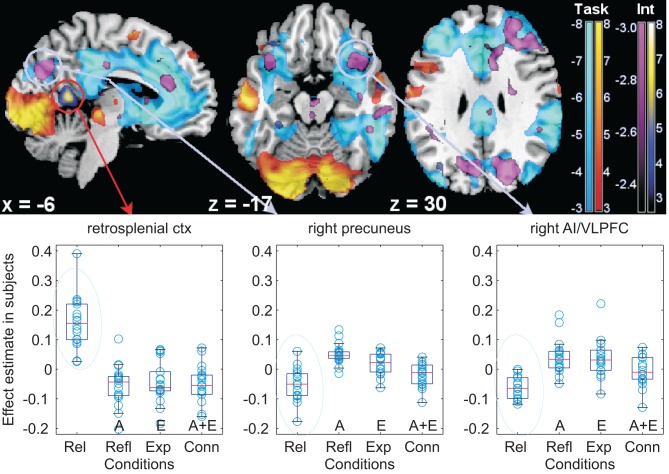
**Interaction between emotional and abstract story versions. Top:** illustration of the spatial overlap between the interaction emotion and abstraction and the deactivations of the main task (light blue). Statistical maps of *t*-values overlaid on a template T1-weighted brain. As in Figure 3, yellow/orange denotes task activations, light blue deactivations relative to fixation, thresholded for display purposes at *p* = 0.005, uncorrected. The interaction is in yellow/blue or in violet (according to its direction). These areas may be compared with those of the regression on individual differences of Figure 3, especially for the medial aspect of the brain on the left, and the orbitofrontal cortex. **Bottom:** box-plots of signal differences between conditions. The light blue circles highlight the textual version where neither abstract nor emotional words were used, and which drives the interaction. For an explanation of the symbols of the box-plots, see the legend of Figure 3. AI/VLPFC: anterior insula/ventrolateral prefrontal cortex; ctx: cortex; Int: interaction.

Finally, we looked at the effect of the interaction between emotional and abstract material in the whole brain (Table 7). A locus in the retrospenial cortex reached significance outside the areas of activation due to reading (Figure 5, red circle, *x*, *y*, *z*: −6, −52, 10, BA17/30; *t* = 8.12, *p* = 0.02). As is apparent from Figure 5, this interaction was due to increased signal in this region in the textual descriptions in which neither emotional nor abstract markers were present. Interaction between emotional and abstract material was also detected in areas that were deactivated by the main task (Table 7 and Figure 5). The largest effects here were in the precuneus/parieto-occipital sulcus bilaterally (BA7; in the main task, the deactivation was *t* = −7.90, *p* < 0.001), in the middle frontal gyrus (BA46/9; in the main task, the deactivation was *t* = −10.15, *p* < 0.001) and bilaterally in the anterior insula/VLPFC (BA38/48; in the main task, the deactivation was *t* = −11.03, *p* < 0.001 on the right; on the left, *t* = −9.18, *p* < 0.001). *Post-hoc* analysis of these whole-brain interactions revealed them to be driven prevalently by the lack of emotional or abstract information, rather than the simultaneous presence of both. In correspondence of the areas deactivated by the task, the deactivation was most pronounced when neither emotional nor abstract words were used in the textual narratives (see the box plots of Figure 5).

**Table 7:** **Interaction between emotion and abstraction, whole brain analysis**.

**Brain region**	**MNI Coord. (mm)**	***t***	***p* (uncorr.)**	***p* (FDR corr.)**	**Cluster size (voxels)**	***p* (cluster corr.)**
L Precuneus/calcarine (BA17/30)	−6, −52, 10	8.12^*^	<0.001	0.022	449	0.009
L Fusiform (BA20)	−30, −30, −24	5.62	<0.001	0.087	126	>0.5
L Inferior frontal triangularis (BA45)	−48, 30, 18	5.20	<0.001	0.143	70	>0.5
L Middle temporal (BA21)	−56, −48, −2	4.40	<0.001	0.314	97	>0.5
L Middle occipital (BA39)	−36, −70, 28	3.77	0.001	0.494	67	>0.5
R Cuneus/precuneus (BA7)	10, −66, 36	−7.38^*^	<0.001	0.070	548	0.003
L Cuneus/precuneus (BA7)	−6, −70, 30	−6.15^*^	<0.001	0.100	326	0.04
L Ant. Insula/Orbitofr. Inf. (BA38/48)	−38, 20, −14	−4.27^*^	<0.001	0.130	406	0.02
L Ventr. Striatum	−14, 12, −4	−5.08^*^	<0.001	0.122	s.c.	
R Ant. Insula/Orbitofr. Inf. (BA38/48)	34, 18, −18	−5.82^*^	<0.001	0.108	772	<0.001
R Caudatus	18, −4, 18	−3.80	0.001	0.135	81	>0.5
L Caudatus	−18, 4, 16	−5.04	<0.001	0.122	184	0.31
R Middle frontal (BA46/9)	24, 46, 30	−5.02^*^	<0.001	0.122	847	<0.001
R Orbitofr. Sup. (BA11)	20, 56, −8	−4.32	<0.001	0.130	85	>0.5
L Anterior cingulum (BA32)	−10, 26, 24	−4.09	<0.001	0.130	170	0.38
R Lingula (BA18)	20, −84, −2	−3.99	<0.001	0.130	204	0.23
R Angular gyrus (BA40)	32, −48, 38	−3.87	0.001	0.133	101	>0.5

Abbreviations: L left R right BA Brodmann area MNI Montreal Neurological Institute p (uncorr.) significance levels, uncorrected p (FDR corr) significance levels at voxel level, false discovery rate correction p (cluster corr.) significance levels cluster-level correction.

Cluster size in voxels of 2 × 2 × 2 mm. The asterisk“

* ” marks results significant at FDR voxel level or cluster level, p ≤ 0.05. Reported are clusters of extent of 50 voxels or larger. s.c.: same cluster.

By comparing Figures 3 and 5, a considerable overlap is apparent in the precuneus and anterior insula/VLPFC between areas affected by the interaction and those modulated by the individual differences in the tendency to use emotional words in later recounting the stories in writing.

## Discussion

We will first comment on the areas modulated by the presence of abstract and emotional material in the narratives and the associated individual differences, and then consider the significance of the findings as a whole.

### Abstraction

When examining the differential activation induced by abstraction, we found the expected increased bilateral activation of DLPFC that accompanies text processing of increased complexity (Just et al., 1996), modulating the activation associated with the task as a whole. In the whole brain analysis, the presence of abstraction was associated with activation in the dorsal medial prefrontal cortex, an area generally linked with difficulty in cognitive processing (Botvinick et al., 1999; Bush et al., 2000). In our data there was also evidence for activation associated with the presence of abstract words in the right hemisphere, most notably in an area across both the temporal and the parietal lobes. However, no correlates of individual differences in abstraction in recalling the stories could be detected in either the ROI or full-brain analysis.

### Emotion

The presence of emotional material elicited activations in the amygdala, as in prior investigations (Ferstl et al., 2005; Kensinger and Schachter, 2006; Costafreda et al., 2008; Yoshimura et al., 2009). The presence of emotional terms was also associated with increases in prefrontal areas, especially in the anterior insula/VLPFC region, which have been reported in previous neuroimaging studies of exposure to verbal and textual material of emotional connotation (Osaka et al., 2004; Ferstl et al., 2005; Kuchincke et al., 2005; Kensinger and Schachter, 2006). These areas are generally associated with semantic retrieval and selection and working memory tasks (Badre et al., 2005). The anterior insula/VLPFC has also been associated with emotion regulation (Ochsner et al., 2002; Ochsner and Gross, 2005; Phan et al., 2005; Kalisch et al., 2006; Wager et al., 2008). Because of the possible regulatory nature of the implicated areas, these activations are consistent with an attempt of participants to control emotion aroused by the textual description of these scenes. However, we could detect no modulation of the signal in these putatively regulatory areas when regressed on the individual propensity to use abstract or emotional words. Rather, less frequent use of emotional words loaded bilaterally on a separate area located more ventrally in the inferior prefrontal gyrus/anterior insula, on the border with the orbitofrontal cortex.

In the prefrontal lobes, emotional material also elicited a relative increase of signal levels in the medial frontal cortex (BA 9-10) extending to the anterior cingulate cortex (ACC, BA32). Several studies have reported the activation of these areas in the presence of emotional material processing (Phan et al., 2004), such as reading emotional narratives (Mano et al., 2009), reading sentences with affective semantic content with or without affective prosody (Beaucousin et al., 2007) exposure to emotional words (Kensinger and Schachter, 2006; Yoshimura et al., 2009), listening to emotional stories (Ferstl et al., 2005), and generating words with emotional connotation (Cato et al., 2004). In our study, this region, which was mostly deactivated by the task, was associated with individual differences in the use of emotional words in recounting the textual description of the scenes. Individuals using less emotional words when recounting the stories in writing deactivated less in the perigenual and retrosplenial cortex when presented with emotional material than individuals with high use of emotional words, who deactivated most in this area. There were therefore two aspects distinguishing these areas from those associated with explicit control: the fact that they were deactivated by the main task, and that they correlated with individual differences in the access to emotional expression in the absence of any explicit instruction in this respect.

### Interaction between abstraction and emotion

The interaction between the abstraction and emotion factors had the purpose of identifying possible networks specifically involved in the elaboration of the combination of both. In the ROI analysis, there was some evidence that this interaction modulated prefrontal cortex activation associated with the task in the MLPFC. This area, which was also modulated by emotional material, is often implicated in imaging studies of semantic processing (Binder et al., 2005). Left MLPFC (BA44-45) and VLPFC (BA47) may mediate a mechanism of selection of relevant information from among competitors and retrieval of knowledge stored in temporal semantic areas (Badre et al., 2005). The tendency of the interaction to be located anteriorly to the effect of emotional markers alone, evident in Figure 2, is consistent with a rostro-caudal gradient of increasing complexity in the prefrontal cortex (Christoff and Gabrieli, 2000; Badre and D'Esposito, 2009).

In the whole-brain analysis the interaction of emotion and abstraction was driven by the simultaneous lack, rather than presence of emotion, and abstraction. Lack of these markers was found to be associated with increased signal in retrosplenial cortex, an area associated with encoding episodic memory and emotion processing (Maddock, 1999; Binder et al., 2005; Buckner and Carroll, 2007; Vann et al., 2009) and implicated in emotion regulation (Wager et al., 2008). Selective activation of precuneus and retrosplenial cortex has also been reported in participants who had been instructed to distance themselves from the emotional content of the stimulus set as a strategy for control (Koenigsberg et al., 2009). Furthermore, the interaction was detected in the anterior insula/VLPFC, which was implicated in studies in which participants had been instructed to suppress emotional expression (Goldin et al., 2008; Wager et al., 2008). The role of these areas in emotion regulation is consistent with the detection in our study of considerable individual differences in these regions, correlating with using less emotional words at later recounting.

## General discussion

In this study we examined the neural correlates of textual markers in narratives concerning themes of emotional importance, motivated by research that has attempted to demonstrate associations between markers contained in narratives and attachment patterns, clinical pathology, or progress in therapy (Bakermans-Kranenburg and van IJzendoorn, 2009). An issue was the extent of which networks that are associated with top–down cognitive control would correlate with these markers. As a whole, the presence of emotional linguistic markers was effective in modulating the signal in areas activated by the task, as demonstrated by the functional ROI analysis. In the prefrontal cortex, we found that activation in the mediolateral and ventrolateral prefrontal regions were associated with individual differences in the frequency of emotional terms in recalling the textual descriptions of the scenes, and with textual narratives characterized by the absence of emotional or abstract terms. However, a finding of our study was that prominent associations with textual markers and individual differences in their use were found outside the main task activations. In some cases, as in the lateral prefrontal cortex (MLPFC and VLPFC), modulation of signal by textual markers resulted in a parcellation of the region into small areas preferentially and separately activated by emotion, abstraction, their interaction, or individual differences in the use of emotional terms. This may be due to a fine specialization of this area (Badre et al., 2005; Badre and D'Esposito, 2009), high individual variability of anatomical regions (Saxe et al., 2006), or a combination of both.

In other cases, the modulation by textual markers concerned areas deactivated by the task. This finding should be viewed with caution, since the present study was not designed to detect deactivations through quantitative estimates of the physiological effects of brain activity. Nevertheless, the observation that prominent neural correlates of individual differences in appraisal styles were found in deactivated areas raises doubts about attributing them to attentional processes recruited as part of top–down control. In this respect, an interpretation based on subclassifying areas according to increasingly finer specializations competes with viewing activation and deactivation patterns as part of large-scale networks in reciprocal interaction, as proposed in other studies (Fox et al., 2005), and attributing to deactivations a functional role in allocating attention through mechanisms that do not depend on executive processes (Shulman et al., 2007). Some studies in particular provide evidence for networks distinct from those associated with explicit, effortful control, but active in allocating attention to contents of behavioral relevance, and whose neural signature may be the modulation of deactivations (Shulman et al., 2007; Corbetta et al., 2008; Viviani et al., 2010). These deactivated areas, such as the ones associated with individual differences in the use of emotional words in the present study (perigenual and retrosplenial cortex) are part of the “default system network” (Raichle et al., 2001; Margulies et al., 2007), which may be involved in stimulus-independent processes and motivation (Raichle and Gusnard, 2005; Buckner and Carroll, 2007). Another possibility is that association of individual appraisal styles in these areas was due to differences in activations in the default network system during the fixation pauses between the narratives. In their review of experimental fMRI studies that have found activations in areas normally deactivated by cognitive tasks, Buckner and Carroll (2007) have noted the frequency of tasks that require spontaneous production of thoughts, as elicited when thinking about the future, making hypotheses, or in social cognition and theory of mind tasks. Here, we had unusually evocative scenes which the participants evaluated passively by reading different versions of stories that related to them. It appears therefore entirely possible that the pauses between reading times were the time when participants were led to actively formulate their own thoughts about these scenes. This would also explain the unusually extensive deactivations observed in our data.

It has been noted that the areas that are part of the default system network are often associated with semantic memory (Binder et al., 2009). This functional attribution is consistent with their modulation by the spontaneous use of textual markers in the present study. The modulations of deactivations observed in the present study may be the trace of different activity in these networks (Corbetta et al., 2008), reflecting selection of information through the coordination of distinct networks brought about by contextual preponderance of emotional or abstract terms, or individual differences in the spontaneous propensity to access emotional content, as revealed by their choice of terms in the rendition of the scenes. This alternative interpretive framework is particularly appealing in the light of the fact that only part of the variation in our data correlated markers of emotional control, either in the text as such or across participants characterized by their individual propensity to characterize scenes, with areas that are typically associated with control of executive nature, such as DLPFC and the dorsal ACC, or even with areas that were activated by the main task. Since the present results were obtained in the absence of an explicit instruction to reappraise or modify the experience associated with perceiving the emotional stimulus, they may refer not only to different capacities of participants to recruit top–down control functions to control the emotional experience, but also to a more comprehensive recruitment of processes involved with access to emotional information. Future research may focus on the role of the default network system in spontaneous thought processes, and its modulation through individual differences associated with vulnerability factors or personality traits of relevance for mental health.

### Conflict of interest statement

The authors declare that the research was conducted in the absence of any commercial or financial relationships that could be construed as a potential conflict of interest.

## Figures and Tables

**Figure A1:**
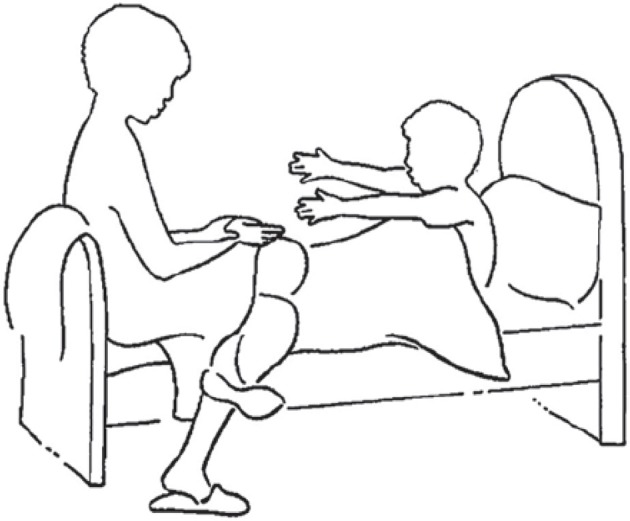
**Carol George, Malcolm West, Odette Pettem (1997).** Adult Attachment Projective. All rights reserved.

## Appendix

### Neuroimaging procedures

All magnetic resonance imaging (MRI) data were obtained with a 3-Tesla Magnetom Allegra (Siemens, Erlangen, Germany) MRI system equipped with a head volume coil. All participants were scanned at the Department of Psychiatry and Psychotherapy III of the University of Ulm. Images were obtained using EPI in transversal orientation (T2^*^-weighted, TR/TE 1540/35, flip angle 90°), of image size 64 × 64 pixels (3 × 3 mm.); slice thickness was 3 mm, with a gap of 0.99 mm. For each volume, 24 slices parallel to the AC-PC plane were acquired for whole-brain coverage. After discarding the first six volumes to allow for equilibration effects, 760 volumes were acquired.

Data were realigned and stereotactically normalized to an EPI template (Montreal Neurological Institute, resampling size: 2 × 2 × 2 mm) using the SPM5 package (Wellcome Department of Cognitive Neurology, London; online at http://www.fil.ion.ucl.ac.uk). All volumes were smoothed using a Gaussian kernel of full width half-maximum (FWHM) 8 mm. Estimates of first level contrasts were brought to the second level to account for subjects as random effects.

### Preparation of textual versions and presentation of stimuli

To determine block length, we conducted a pilot experiment on nine subjects in which data on the time required to read one version of the story were collected. Reading time was similar for all story versions, and averaged 33.4 s with a standard deviation of 2.1 s. Block length was determined as the 85th percentile of these reading times. This procedure ensured that in the large majority of presentations subjects were exposed to new textual material during the block. The timing of the interstimulus intervals (5 s) was subsequently set such that the correlation between the estimates from the contrast regressors (after convolution with the canonical BOLD response function) was less than *r* = 0.01, including the regressors for the factor contrasts and their interaction and the regressor contrasting all reading trials with fixation. We verified that these correlations remained low (less than *r* = 0.05) when weighted by the matrix used to model autocorrelation at the first level. The experiment was implemented with the software “Presentation” version 11.0 (Neurobehavioral Systems, Inc, Albany, CA). Participants viewed stimuli through goggles masking the whole field of vision (Resonance Technology, Inc, Northridge, CA). Text was in white body against a black screen.

### Example of material used in the study

In the standard AAP interview used for clinical or research purposes, participants are asked in a semi-structured format to describe the scene in the picture, and imagine what characters in the scene may be thinking or feeling and what participants believed might have happened or happen next. In the present study, participants were exposed to the AAP scenes, and the relevant stimuli consisted of written accounts of what characters may be thinking or feeling and what might happen in the scene, systematically varying in the use of emotional and abstract words. These accounts correspond to different possible subjective experiences of the same scene. Participants were asked to provide their own account only after the scan, once for each scene. These accounts were scored with the same criteria used to systematically vary the accounts presented in the scanner.

This is an exemplary scene used in the study, followed by the four account versions: with low emotional and abstract usage, with high abstract usage, with high emotional usage, and with high emotional and abstract usage. The English translation is meant to be indicative of the general tone of the German original, and tends to be more literal than a professional translation would ordinarily be.

1. Niklas ist zwölf Jahre alt, er wird gerade von seiner Mutter geweckt. Er weiß, heute ist der Tag, an dem er mit seinem Vater und seiner Mutter nach Kenia fliegen wird. Die Reise wird ca. sechs Stunden mit dem Flugzeug dauern. Sein Vater hat schon viele Bücher aus der Bibliothek ausgeliehen. Zunächst hat sich Niklas die bunten Bilder von den Giraffen, Löwen, Elefanten und Tigern alleine angesehen. Er hat dann seinen Vater viel gefragt und dieser hat Niklas erzählt, dass es in Kenia immer sehr warm ist. Des Weiteren gebe es dort keinen Winter, aber so was Ähnliches, was man als Regenzeit bezeichnet. Der Vater erklärt, dass Kenia größer ist als Deutschland und dass dort nicht alle Kinder zur Schule gehen können. Während des Schlafs hat Niklas von den Tieren und Pflanzen geträumt.
  Niklas is 12 years old, and is just being woken up by his mother. He knows that today he will be flying to Kenya with his father and mother. The flight will last more or less 6 h. His father has already borrowed several books from the library. Niklas first looked at the coloured photos of giraffes, lions and tigers on his own. Then he asked a lot of questions to his father, who told him that in Kenya it is always hot. Moreover, there is no winter there, but something that people call rain season instead. The father explained that Kenya is larger than Germany, and that not all children are able to go to school. While asleep, Niklas dreamed animals and plants.
2. Niklas ist zwölf Jahre alt und wird von seiner Mutter geweckt. Er hat Ferien und weiß, sie werden heute eine weite Reise in ein fernes Land antreten. Seine Eltern haben Kenntnisse von vielen Regionen der Welt. Insbesondere bereisten sie einige Nationen in den Tropen und haben viele Erfahrungen mit den Menschen und ihren Bräuchen und Sitten gemacht. Mit seinen Eltern hat er sich bereits viel Literatur über diese Gebiete ausgeliehen. Darin waren viele Bilder über Landschaften der Länder Afrikas zu sehen. Diesmal wird die Familie das erste Mal zusammen eine Abenteuerreise nach Afrika antreten. Niklas erschienen im Traum Bilder über die Weiten Afrikas, selbst die Gerüche und Farben der Blumen konnte er wahrnehmen.
  Niklas is 12 years old and is being woken up by his mother. He is on holiday, and knows that today they will leave for a long trip in a distant land. His parents have knowledge of a lot of regions of the world. Above all, they have traveled in tropical countries, making experiences of their human beings, their customs, and lore. With his parents Niklas has already borrowed a vast literature on these territories. In this literature there were lots of images to see of landscapes of the countries of Africa. This is the first time that he and his family undertake an adventure journey to Africa together. To Niklas, images of the vastness of Africa appeared in his dreams; he could even perceive the smell and the colors of the flowers.
3. Niklas, ein zwölfjähriger Junge, wird von seiner Mutter am Morgen sanft geweckt, er erwacht glücklich und umarmt sie. Er weiß, heute wird er mit seiner Mutter und seinem Vater zusammen nach Kenia fliegen. Sicher wird das lange Sitzen im Flugzeug etwas anstrengend werden, aber er freut sich riesig auf die schönen Pflanzen und die wundervollen Tiere, die er nur aus dem Zoo kennt. Mit seinem Vater hat er gemeinsam viele spannende und interessante Bücher aus der Bibliothek ausgeliehen. Sehnsüchtig und ungeduldig hat er diesen Tag herbeigesehnt. Sein Vater und seine Mutter kennen sich ausgezeichnet mit Afrika aus. In der Nacht hat Niklas einen wundervollen Traum gehabt, er sah die prächtigen Pflanzen und konnte exotische Tiere schreien hören.
  Niklas, a child of twelve, is being gently woken up by his mother in the morning. He wakes up with joy and embraces her: He knows that today he will be flying to Kenya together with his mother and his father. Surely, the long seating in the airplane will be a bit tiring, but he is really keen on seeing the beautiful plants and the wonderful animals that he has known only from the zoo. Together with his father he has borrowed from the library a lot of exciting and interesting books. He has yearned for this day impatiently. His father and his mother already know Africa well. During the night Niklas had a marvelous dream. He saw wonderful plants and heard the exotic animals howl.
4. Niklas, ein zwölfjähriger munterer Junge, wird zärtlich am Morgen von seiner Mutter geweckt. Er wird heute mit seinen Eltern eine anstrengende, aber sehr interessante Reise in ein fernes, großartiges Land antreten. Mit großen Erwartungen hat Niklas seine Ferien herbeigesehnt. Seit längerer Zeit hat er an den Wochenenden neugierig viel Reiseliteratur gelesen. Diese Nacht hatte er einen wundervollen Traum. Er fuhr durch eine geheimnisvolle Landschaft, die ihn ungewöhnlich mit voller Macht anzog. Er sah große Palmen und faszinierende, gefährliche Wesen. Intensiv nahm er auch den starken Geruch der Pflanzenwelt und die unheimlichen Schreie der Tiere wahr. Als ihn die Stimme seiner Mutter am Morgen weckt, wirkt dies wie eine sonderbare Verwandlung.
  Niklas, a lively child of twelve, is affectionately waked up by his mother in the morning. Today he will be leaving with his parents for a tiring but very interesting trip in a distant and exciting land. Niklas has been looking forward to this holiday with big expectations. For many week-ends his main interest has been reading a lot of travel literature. Last night he had a wonderful dream. He went through a mysterious landscape that made him feel unusually full of strength. He saw big palms and fascinating, dangerous beings. He also perceived the strong smell of the plants, and the uncanny howl of the animals. When the voice of his mother waked him up in the morning, he had the sensation that things changed back to reality.
